# Including ‘Work as a Treatment Goal’ in the Care for Patients with Chronic Diseases: The Development of a Generic Care Model—A Descriptive Study

**DOI:** 10.1007/s10926-024-10215-w

**Published:** 2024-06-19

**Authors:** Desiree J. S. Dona, Marlies E. W. J. Peters, Theo F. Senden, Sjaak Bloem, Herman Bartstra, Marieke T. Jacobs, Frederieke G. Schaafsma, Patrick Jeurissen

**Affiliations:** 1 Department of Primary and Community Care, Radboud University Medical Center, Geert Grooteplein 21, 6525 Nijmegen, EZ The Netherlands; 2 Center for Marketing & Supply Chain Management, Nyenrode Business University, 3621 Breukelen, BG The Netherlands; 3 Department of Public and Occupational Health, Amsterdam UMC, Amsterdam Public Health Research Institute, Amsterdam, The Netherlands; 4 Department of IQ Health, Radboud University Medical Center, Geert Grooteplein Zuid 10, 6525 Nijmegen, GA The Netherlands

**Keywords:** Chronic illness, Quality of life, Quality of work, Work as a treatment goal, Clinical occupational physician, Personalised care, Work-oriented medical care

## Abstract

**Background:**

The Netherlands faces 60% prevalence of chronic conditions by 2040, impacting societal participation and quality of life. Current clinical care inadequately addresses these consequences, and most hospitals do not integrate occupational health in their care.

**Objectives:**

To develop a generic person- and work-oriented medical care model (WMCM) based on real life experiences with work-oriented care and supporting the chronically ill in active societal participation.

**Methods:**

A qualitative research project with a participative approach in one hospital (November 2019 until March 2020). In an expert meeting, a schematic representation of a work-oriented care model was developed. Subsequent discussion rounds, with professionals from different patient groups, iteratively refined the model to a WMCM.

**Results:**

Consensus was reached after seven rounds of discussion, defining the model’s core elements (1) a combination of biomedical and biopsychosocial approaches, (2) involvement of a clinical occupational physician in the treatment team, (3) a coordinating role for nursing specialists, and (4) incorporation of a work-oriented intervention plan (WoIP) into the treatment plan. Advocating early attention to societal participation, the model emphasises the WoIP and consensus on monitoring indicators. The final goal is a sustainable return to societal participation, considering both quality of life and work.

**Conclusion:**

It is feasible to develop a generic person- and work-oriented care model for patients with chronic illness within a hospital care setting. Collaboration between healthcare professionals and a specialised occupational physician, with a central role for nurses, is deemed crucial.

## Background

The prevalence of people in the Netherlands with at least one chronic disease, including chronic consequences of treatment will increase to 60% in 2040 [1]. Currently, 20% of the potential workforce (18–67 years) is suffering from one or more chronic conditions. Thus, this number will increase substantially due to ageing, postponed retirement age, and improved care [2–4]. A major proportion of people with chronic illness experience problems with keeping their jobs or returning to the labour market [5]. Not having a job has significant adverse effects on quality of life and income [6].

The employment rate of people with chronic diseases in the Netherlands is approximately equal to the average of all member states of the organisation for economic cooperation and development (OECD) [4]. The OECD data show that Sweden, Denmark and Germany have been able to reduce unemployment amongst the people with a disability, this is in contrast to the Netherlands, where the gap between the healthy and the people with a chronic disease in terms of societal participation has actually increased in recent years [7].

Being able to continue working despite chronic illness has a strong and positive impact on mental and physical health, self-esteem, perceived control of life and the experience of happiness [8–10]. A substantial portion of people with chronic illnesses indicate that work is a priority in their lives and that they need tailor-made, personalised support to maintain or return to work as quickly as possible and in a sustainable manner [11–17].

Participation in the labour force also contributes to lower costs for healthcare, less absenteeism and less disability benefits. A job brings structure to daily life, ensures confidence and provides more social contacts [10, 14, 16, 18–20]. In the Netherlands, the effects of chronic illnesses on social costs, including healthcare and social benefits are estimated to be 30 billion euro per year [4].

Interventions have been developed to improve the participation of the chronically ill [21–24]. The focus of these support activities can be different. There are patient-oriented (e.g. self-management programmes or rehabilitation programmes) and workplace-oriented interventions (e.g. instruments for training and adaptation of the work (place)). Most interventions focus mainly on changes in work (place). Work-oriented medical care is not common yet and there is little literature available on this subject. The few clinical interventions do focus mainly on one-off support and/or referral to an expert [21, 23, 24]. Due to varying effects of the tested interventions there is reason to conduct further research. Several reports have recommended paying more attention to work participation in regular health care. For example, work problems can be discussed at an early stage and throughout the patient journey in the doctor’s office, because patients with chronic illness regularly visit their general practitioner and medical specialist [4, 25–28].

Maintaining work can be seen as an important aim of health care that requires attention and should therefore also be a treatment goal in the care of chronically ill patients [25]. However, literature focussing on the integration of work-related components into clinical care appears to be limited [23, 24, 27–31]. Previous Dutch projects aimed at behavioural change amongst medical specialists and improving the cooperation between regular and occupational care have not led to structural improvements [4, 25, 26]. It is important that a method is developed in which work is included as a treatment goal of good care.

This prompted the development of work-oriented care in the daily care practice of some patient groups in a Dutch university hospital, starting from 2016. This study describes the work-oriented care that was continuously developed over the years. We used oncological care as a testbed to describe the contours of such a care model, because of long term experience and the extensive patient group, after which we broadened the model to other patient groups. A twofold research question was formulated: (1) Can work-oriented care be integrated in regular oncological care and transformed to a generic care model, also for patients with other chronic diseases? And (2) which are the important indicators and outcome measures, that also can be applied to other diseases?

## Objectives

To develop a work-oriented medical care model (WMCM) to improve care for chronically ill patients who are able and wish to participate in society in which the goals of these patients are taken into account from diagnosis onwards.

### Methods

We used a qualitative research design with one expert group meeting followed by discussion rounds with experts [32]. Also some elements of participatory action research were used; (1) the work-oriented care was developed in a participatory manner at the request of healthcare professionals and patients and (2) the first author of this paper works as a clinical occupational physician (COP) in oncology and participated in both the expert group meeting and in the discussion rounds.

An expert group meeting was held in order to arrive at a schematic representation and description of the WMCM in clinical oncological care. Then, in discussion rounds with other experts, insight was gained from their daily care practice, and through an iterative process, the schematic care model for oncology was made generic to also address the needs of patients with other chronic diseases. Discussion rounds with experts took place until saturation was reached, see Table 1.

**Table 1:** Overview of methods

Period	Methods	Participants	Subjects and themes
Nov 2019	Expert group	6 participants all involved in oncological care	(1) Medical and occupational health perspectives on phases in oncological treatment (2) Referring patient to COP (3) Cooperation between medical team and COP (4) The work-oriented care path (5) Care of the COP
Nov 2019–October 2020	2 discussion rounds	1 researcher and 2 COPs: 1 COP (A) involved in the oncological care, 1 COP (B) involved in another patient group	Medical and occupational health perspectives on phases in treatments for different diseases
	2 discussion rounds		Indicators and outcomes that could be added to the schematic generic care model
	3 discussion rounds		Create a schematic overview

*COP* clinical occupational physician

#### Expert Group

The expert group meeting was held in November 2019. The group consisted of six participants (i.e. two COPs oncology and neurology, one oncologist, one nursing specialist oncology, one researcher and a representative from the patient organisation). All participants were involved in a forerunner model of work-oriented care that had been developed at the request of patients and professionals based on experiments in daily practice in this hospital in the previous years. The expert group session used a stepwise manner in which there were five rounds moderated by an independent professional moderator, see Table 1.

On the basis of case vignettes, five different themes were discussed: (1) Which phases can be distinguished in oncological treatment from a medical and occupational health perspective? (2) Who refers and what are the reasons for referring patients to the COP? (3) How is the cooperation between health care professionals and the COP? What is the role of the multidisciplinary team (MDT)? At what point in the oncological care pathway is a patient referred to the COP? (4) What does the work-oriented care path look like? (5) What would the care of a COP add in the care of the patient?

After each round, the moderator noted the findings. The care model was then constructed schematically in a plenary session and adapted after each round of discussions.

#### Expert Discussion Rounds

Three discussion rounds took place face to face over a four-month period, from November 2019 to March 2020. Due to the COVID-19 pandemic, the other four discussion rounds were conducted online in the period March to October 2020. In each round the same two COPs, the researcher and the moderator were involved. To generalize the schematic care model, a COP with experience in work-oriented care from other patient groups (neurology and cardiology) also participated. These patient groups were chosen because practical experience was also gained here. Themes and questions were brought in and discussed by the participants in each discussion round. The moderator’s follow-up consisted of asking in-depth questions until agreement was reached. After each discussion round, the moderator made adjustments to the schematic representation of the care model. The first two rounds focused on understanding whether different phases could be distinguished from the medical versus occupational health perspective. It was also discussed whether these phases may differ depending on the patient group. The purpose of the third and fourth discussion round was to reach consensus on the desired indicators and outcomes that could be added to the schematic care model. In the final three discussion rounds, the goal was to create a schematic overview in which all components could be displayed in an integrated and logical manner, including feedback loops.

During all discussions, the schematic representation of the care model was re-evaluated on the basis of new case vignettes. This led to adjustments in the representation and indicators of the care model. The new case vignettes related to various patient groups (Young Stroke patients, patients with hemato-oncological diseases, patients with Parkinson’s disease as well as patients with congenital heart disorders) to ensure that the schematic representation of the care model would become more generic.

#### Data Analysis

Based on all data collected, the work-oriented care model in oncological care was presented schematically. Before the start of each discussion round, themes and topics were appointed and selected for further deepening in further discussion rounds by the 2 COPs and the researcher based on their knowledge and practical experience. Themes and topics were abstracted into a schematic representation of a WMCM by asking in-depth questions, discussion and analysis of experts.

## Results

### Expert Group

Table 2 presents an overview of all discussion rounds, the topics discussed, the time required and the results.

**Table 2:** Overview of expert discussion rounds

Duration of the rounds	Themes	Results
Round 1: 0,5 h	Medical and occupational health perspectives on phases in oncological treatment	Medical perspective: phases in a fixed pattern Occupational health perspective: a less strict sequence of phases
Round 2: 0,5 h	Referring patient to COP	The patient reference to the COP is made by MS, NS and MDT Different reasons for referring
Round 3: 0,5 h	Cooperation between medical team and COP	Work is discussed during the diagnostic phase In the MDT cooperation is important At any time patients can be referred to the COP Healthcare professionals experienced a lack of knowledge about occupational health Ideally, attention should be paid to (societal) participation from diagnosis
Round 4: 0,5 h	The work-oriented care path	Work-oriented care should be integrated and tailored to the patient The WoIP is a dynamic process
Round 5: 0,5 h	Care of the COP	Timely information and education empowers and support the patient to manage its situation and make informed decisions Occupational health knowledge in the MDT Sometimes no return or only partial return to work is also a good outcome measure The WoIP should aim experiencing quality of life (QoL) and quality of work (QoW)

*COP* clinical occupational physician, *MS* medical specialist, *NS* nursing specialist, *MDT* multidisciplinary team, *WoIP* work-oriented intervention plan, *QoL* quality of life, *QoW* quality of work

#### First Round

It was discussed what phases can be distinguished in the treatment for an oncological disorder. From a medical perspective, the patient goes in through several consecutive phases: diagnosis followed by treatment, follow-up, and in some cases screening for late effects. From the occupational health perspective, such strict sequence of phases appeared less evident, as health aspects, personal and environmental factors can give rise to concerns or problems regarding work and income at any stage of treatment.

#### Second Round

In the second round the referral process to the COP was discussed. Participants reported that referral usually was made through the medical specialist (MS), the nursing specialist (NS) or the multidisciplinary team (MDT). The role of the NS as a central care provider was emphasized by all. Participants indicated that over the years the NSs increasingly referred patients to the COP. It appeared there were many reasons for referrals. For example, when there is a question related to causality; e.g. ‘Can the cancer be caused by risk factors in the workplace?’ or ‘Were there risks in advance for work resumption due to, for example, pre-existing health problems, personality characteristics, pre-existing absenteeism or due to illness and treatment?’.

Or when a question is related to the intervention; e.g. ‘What are the effects of treatment on work outcomes, and are there any other treatment options?’.

Another reason for referral is when the patient experiences problems with work or income and is in need for support. Negative expectations of both the patient and the care professional about return to work can also be a reason for referral to the COP. And finally, it was mentioned that if the patient wants to work (partly) during treatment this is also a reason to refer to the COP. It was stressed that each patient can have their own wishes and preferences for treatment as well as for goals in work. In any of the phase, situation and/or preference can lead to a request for help from the patient.

#### Third Round

In the third round, the cooperation between the healthcare professionals and the COP was subject of discussion. First, the timing of referral to the COP was discussed. The MS and NS indicated that they already ask the patient during the diagnostic phase about the work status and goals or wishes. At this point information on the support and working method of the COP is provided. The MS, NS and the MDT are aware that the patient can be referred at any time. The expert group gave extensive consideration to the positive effects on the maintenance of working capacity, and to the fact that loss of work and income can be significant if they are not addressed in a timely manner and vice versa. The healthcare professionals experienced initially a lack of knowledge about the (legal) frameworks and the social map of aid workers and bodies. This was an obstacle, but experience, especially the discussions of case histories, gradually improved this gap. In addition, all members agreed that ideally attention should be paid immediately to (social) participation from diagnosis. All expert group members also agreed that the function of the MDT is important for discussing case studies and cooperation.

#### Fourth Round

In the fourth round, aspects of work-oriented care were discussed in more detail. The COPs contributed to a work-oriented intervention plan (WoIP) in consultation with the patient. The COP does an intake and makes a multifactorial problem analysis for drawing up a WoIP. The COP lists facilitators and barriers for returning to or retaining work. Together with the patient, the COP makes an intervention plan, geared to achievable goals and patient wishes, with advice and interventions for the healthcare, work and/or social domains. There was consensus on the importance of incorporating a WoIP into the treatment plan. The COPs indicated that it is sometimes necessary to test the goals of the patient for feasibility. This often requires coordination with the MS, NS and the MDT, and also with the professionals in the occupational health field, although this is not always possible. This coordination is a dynamic process. Over time, factors that influence the execution of the WoIP may change. As the situation may change, it is important that all those involved, including the patient, need to anticipate.

#### Fifth Round

**Fig. 1:**
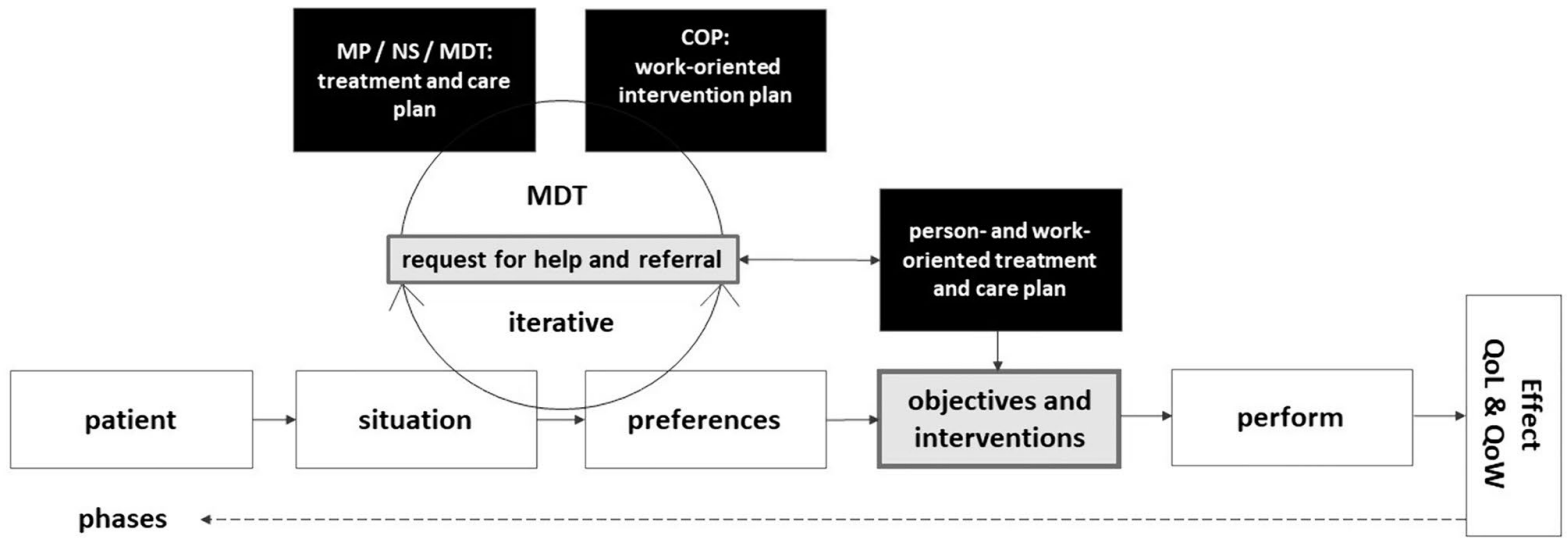
Schematic representation of the care model, after five discussion rounds

In the fifth round it was discussed what the COP adds in the care of the patient. This extensive discussion showed that if the patient receives timely information and education that empowers, this can help them manage their situation and is supportive when making informed decisions. The healthcare professionals indicated that by discussing the patient problems with the COP in the MDT, they also gained more knowledge about work-oriented care. As a result, health care professionals felt more able to provide basic care on work and income, and felt more confident to recognise when and why they should refer to the COP. The role of the MDT proved to be important for both discussion of case histories and cooperation in work-oriented care. It also proved to be a good place to evaluate and adjust everyone’s input into the care model. Finally, another important conclusion was that not all cases involve rapid and/or full return to work, but that sometimes no return or only partial return to work is a good outcome. The starting point of this model focuses on a sustainable and healthy return to social participation, with a stable income for the patient. It was agreed that all interventions should aim for both optimal quality of life (QoL) and work (QoW), see Fig. 1.

### Legend

White blocks: information obtained from the patient.

Black blocks: information obtained from the healthcare professional(s).

Grey blocks: information obtained by shared decision-making.

### Expert Discussion Rounds

Table 3 presents an overview of all discussion rounds, the subjects and themes discussed, the time required and the results. After seven discussion rounds, saturation of information occurred and the schematic representation of the WMCM was considered complete.

**Table 3:** Overview of discussion rounds

Duration of discussion rounds	Subjects and themes	Results
Discussion rounds 1 and 2: 2 meetings of 1 h	Medical and occupational health perspectives on phases in treatments for different diseases	(1) In the biomedical approach a treatment plan is based on medical guidelines and is personalised. In the biopsychosocial approach a multifactorial problem analysis based on the ICF method is used for drawing up a WoIP (2) The work-oriented care model should be flexible
Discussion rounds 3 and 4: 2 meetings of 1 h	Indicators and outcomes that could be added to the schematic generic care model	The conceptualisation provided in the ICF makes it impossible to understand disability without consideration and description of the environmental factors
Discussion rounds 5, 6 and 7: 3 meetings of 1 h	Create a schematic overview	See Fig. 3

*COP* clinical occupational physician, *MS* medical specialist, *WoIP* work-oriented intervention plan, *ICF* international classification of functioning

#### Results of the First Two Discussion Rounds

**Fig. 2:**
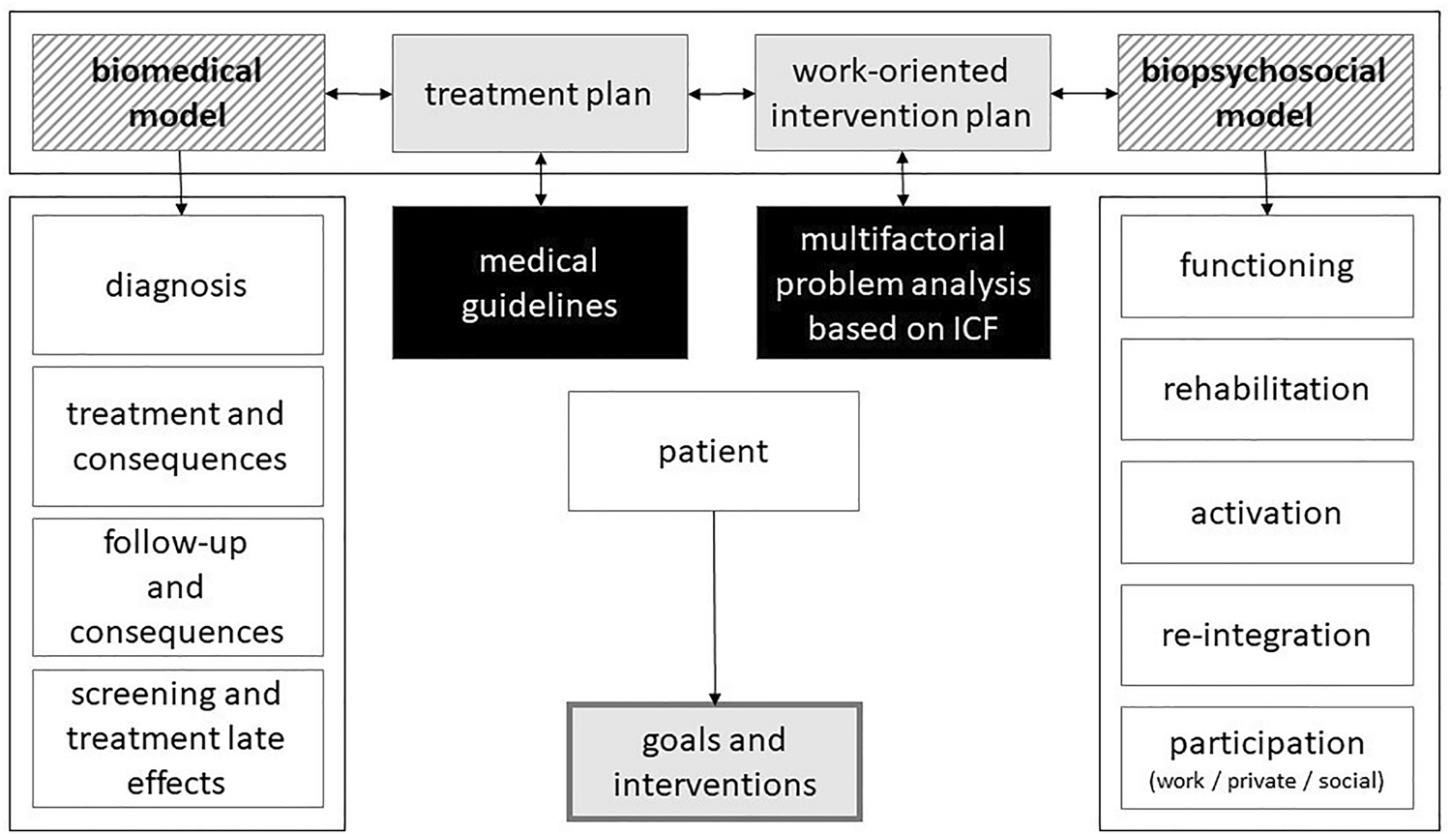
Specific components and different phases from the perspectives of treatment and work

From the perspective of the MS, the patient has a question regarding diagnosis, treatment, consequences, and/or late effects. This is appropriate to the medical specialist’s biomedical thinking framework, see left side Fig. 2. However, a patient may also have questions regarding the effects and consequences of the disease and/or treatment for work. To provide appropriate care for such requests, a biopsychosocial approach is necessary. This approach is one of the thinking and working methods of the COP, see right side Fig. 2.

For patients, there is no strict order in when phases occur over time. They can change randomly due to changes in their situation. These changes can be medical, but they can also be in the personal sphere. Each phase, from both the biomedical and the biopsychosocial approach, has its own needs and consequences and requires a personal approach. According to the MS and COP, this leads to an important conclusion for work-oriented care: this care model should be flexible so that tailor-made care for each patient can be guaranteed regardless of the nature or phase of the disease or the personal and private circumstances of the patient. In the biomedical model a treatment plan is based on medical guidelines and is personalized. In the biopsychosocial model the COP composes a multifactorial problem analysis based on the ICF method (International Classification of Functioning, Disability and Health) to suggest a WoIP. The specific components and the phases from the perspectives of both treatment and work are specified in Fig. 2.

#### Results of the Third and Fourth Discussion Rounds

The COPs who participated mentioned that when work is integrated into healthcare as a treatment goal, they need consensus on indicators and outcome measures to monitor, to evaluate and adjust work-oriented care at the level of the individual patient. In order to reach consensus on the desired indicators and outcomes, the working method of the COP was discussed and clarified. Based on the ICF model, the COP describes functioning from three perspectives: body, person, and societal. The ICF model organises information in two parts. The first part describes functioning and disability, the second part adds the contextual factors. The contextual factors are divided into environmental and personal factors. The conceptualisation provided in the ICF makes it impossible to understand disability without consideration and description of the environmental factors. All factors can affect the patient; and a problem in body function or structure can lead to impairments, limitations in activity and restrictions in participation. The environmental and personal factors are categorised in limiting, hindering or promoting factors.

All factors will affect the WoIP to varying degrees. The COP assesses whether there is a correlation and/or interaction between the various factors. For example: cognitions about experienced health complaints can hinder participation and increase healthcare consumption; lack of support and understanding in the work system can lead to new health problems (e.g. stress or anxiety); and a pre-morbid functional problem can hinder the resumption of work and thereby adversely affect income. Information about these factors can be both expert-driven or a combination of patient/expert-driven.

#### Results of the Final Three Discussion Rounds

In the last three discussion rounds, the goal was to create a schematic overview in which all discussed components are displayed in a comprehensive way, including feedback loops. The situation and preferences of the patient are important starting points for the goals and interventions that are included in the care plan (person-oriented care). Interventions from the COP may include: (1) discussing the consequences of illness and treatment for work, (2) assisting with treatment adjustment, in the interest of maintaining working capacity, (3) explaining relevant laws and regulations, (4) referring to work rehabilitation, (5) referring to other interventions that helps the patient to reduce the distance from the labour market, (6) justification of limited (duration) employability, and (7) consultation and alignment with the work and social domain, including insurance companies.

The patient and professionals work together to achieve the goals of the patient. The COP coordinates and links all factors affecting work, including its social benefits, that are involved transmurally and across the domains in the care network of the patient. The effects on QoL and QoW are evaluated and, if necessary, adapted to the (new) situation. A patient can enter a new phase or stay in the same phase which can require adjustments.

**Fig. 3:**
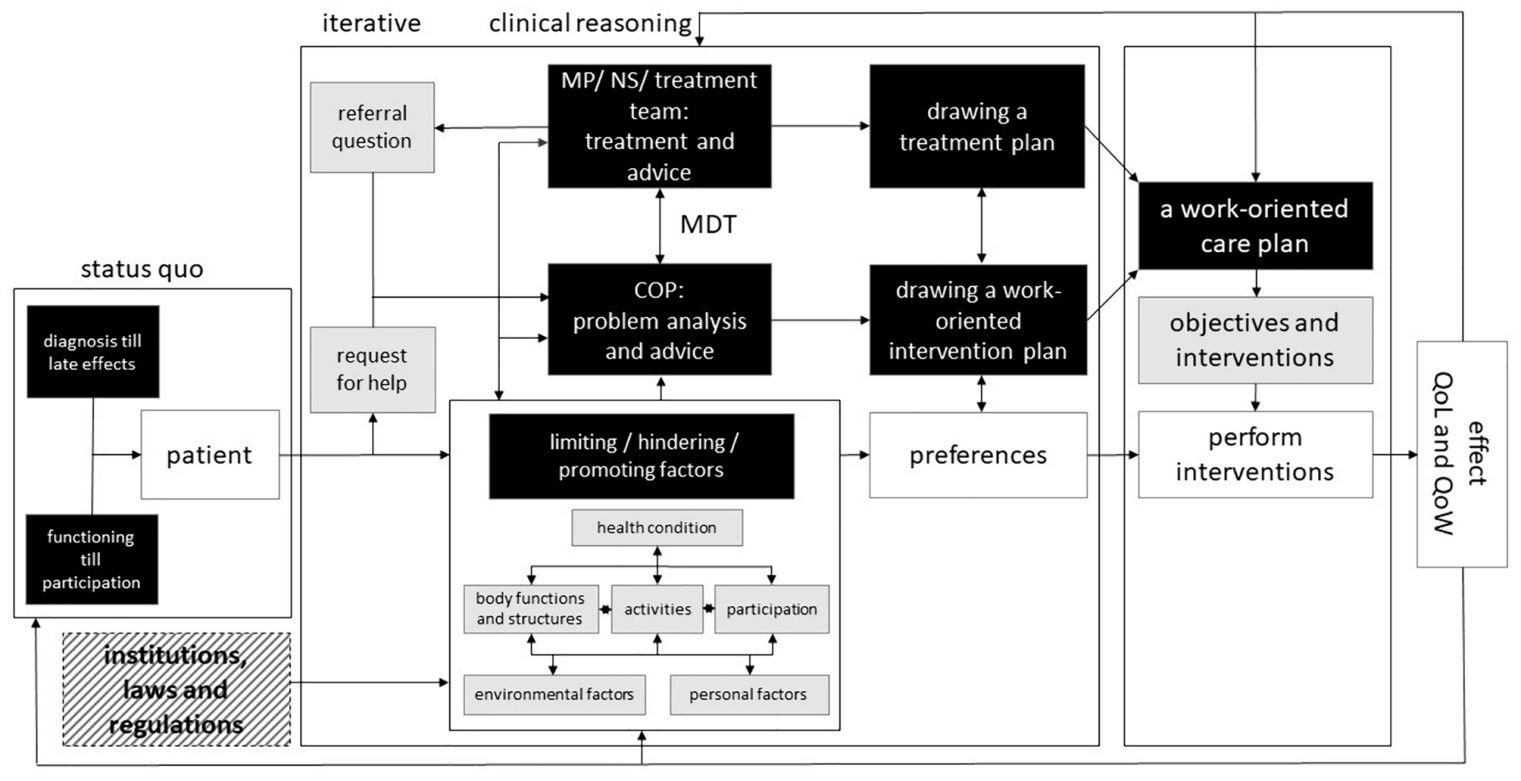
Comprehensive WMCM, including loop feedback

Figure 3 represents an overview that includes all discussed components in an integral way. This figure is a schematic representation of the WMCM as it may have arisen in the context of this specific hospital, namely appropriate to the strategy and objectives of this hospital, person-oriented and integrated in the daily working methods of healthcare professionals in this hospital. A final section was then added to the model to illustrate the influence of socio-political context via relevant institutions, for example the Netherlands Employees Insurance Agency and an occupational health and safety service, legislation and regulations (shaded in grey). These hold direct impact on the situation of the patient. In the current model, these are taken into account by the COP.

## Discussion

This paper describes the development of a WMCM to improve the clinical care for chronically ill patients who wish to participate in society and/or keep their job. We choose for a close collaboration between researcher and healthcare professionals involved in work-oriented care (participative approach) to conceptualise experiences into a WMCM. It was concluded that the focus of work-oriented care should involve the following elements: (1) the goals of the patient are paramount in work-oriented care (person-oriented and tailored care), (2) work-oriented care contributes to healthy and sustainable participation (care based on positive health), (3) the WoIP is part of the treatment plan (integrated care) and (4) factors and indicators relevant to outcome measures will have to be identified.

Over the course of the different discussion rounds it appeared that it was important that the principles and mental frameworks were jointly interpreted in order to be integrated and described in a comprehensive model. Three distinct frameworks were considered important. Firstly, the input of the patient who contributes goals and wishes. Secondly, the input from the medical model including diagnosis, treatment, effects of treatment up to and including late effects. And finally, the input from the biopsychosocial perspective, which sees health as a result of a dynamic interaction between functioning, disease-specific and contextual factors. Strengths of our developed care model are the starting point that goals and wishes of the patient are paramount and the possibility of continuous adjustment of these goals given the changes in the situation of the patient. The new aspect of this WMCM is in particular the integration of a number of aspects. These aspects concern the biopsychosocial approach, the input of occupational health expertise in the treatment team, the explicit attention to work and discussing and including patients’ wishes and goals for work.

This study adds to the literature the description of and support for integrated generic work-oriented care models [29–33]. Our proposed model however, differs from other work-related support interventions in clinical care. First, most of these interventions are designed for specific target groups, for example patients with cancer [24, 30], kidney diseases [27] or rheumatic and musculoskeletal conditions [31]. We know of only one intervention in the Netherlands that focuses on stay-at-work of (self) employed patients with any chronic disease [28]. Second, most literature shows that work-related interventions are designed to refer the patient with work problems to a (singular) work-related intervention, without changing the clinical care pathway [23, 24, 29, 31]. Third, other studies mainly focus on behavioural change of the healthcare professional to discuss the patient’s work problems and refer to an expert in case of complex problems [24, 27, 28, 33, 34] whereas in our model we have opted for a COP as a dedicated discipline in the treatment team that can discuss work and can also connect with occupational healthcare. Fourth, most studies focus on salaried workers [23, 31], whilst our model provides work-oriented care for every chronically ill of working age: students, start-ups on the labour market, salaried workers, self-employed persons, workers with flexible contracts, people on benefits, and informal caregivers. Finally, most studies do focus on job retention or return to work [24, 28, 31, 33]. Our model of work-oriented care can also involve a limitation or even a halt to working in case that work ability is decreasing over time and when this is considered appropriate according to the patient, the COP and health care providers involved. In such cases, the continuous adaptation of work and retention of income and meaningful activities become the treatment goals. Such treatment objectives are also discussed for people who, for medical reasons can hardly participate in paid work, sometimes even at the beginning of working life.

Another aspect of our proposed work-oriented care model is its contribution to the development of a learning system. Because the COP is a member of the treatment team, the COP will acquire more disease-specific knowledge. On the other hand, all team members will gain knowledge and experience with work-oriented care by contributing the COP’s expertise into the treatment team [35–37].

The role of the nursing specialist (NS) as a central care provider was increasingly emphasised. In the treatment, the NS focuses on care that contributes to the health, functioning, quality of life and dignity of the patient. The NS follows the patient journey, and if necessary looks beyond the boundaries of the own institution or organization [36, 38]. The perception was that referrals to the COP were more often from nurses and nursing specialists than from medical specialists. These professionals seem to be able to play a role in triage, monitoring of the intervention process and the patient in achieving their goals for work. Medical specialists may focus more often on the aetiology and treatment of the disease, whilst the nurse(s) (specialists) pay more attention to the effects of the disease and treatment and to the impact on daily functioning and quality of life [36, 38].

The MDT appears to have an important role to play in work-oriented care, although the extent of each person’s actions in relation to work has not yet fully crystallised. For example, it appeared that if a social worker (or another healthcare professional such as a psychologist or occupational therapist) is also a member of the MDT, coordination between the COP, the NS and the social worker is necessary. The COP has medical occupational health knowledge that is necessary for the medical diagnosis, determining the direct consequences for activities and participation—or in other words—work ability, and for drawing up a multifactorial problem analysis in which also external and personal factors are taken into account. The social worker (and other healthcare professionals as mentioned) mainly has a supporting, accompanying and treating role in patient care in carrying out targeted interventions based on the multifactorial problem analysis. Thanks to the cooperation with the COP, we can see that knowledge about the (legal) frameworks and the social map of aid amongst healthcare professionals is increasing. A practical example of the importance and development of work-oriented care in the Netherlands is the desired national approach in the care of AYAs (Adolescent and Young Adults with cancer), in which the COP preferably becomes a permanent member of the care team [39, 40].

This study contributes to conceptual knowledge about integration of work as a treatment goal in clinical care. The development of the WMCM is mainly expert and practice based. Existing knowledge, research and concepts (e.g. the biopsychosocial approach and the ICF model) were indirectly involved because the COPs made important contributions in the development of the WMCM. With qualitative research and a participative approach the tacit knowledge that has been gained through accumulated joint experiences has been made explicit by structured exchange and wrap up in multidisciplinary groups of health care professionals.

## Methodological Considerations

### Strengths

This study has a number of strengths. First of all, the model of work-oriented care is developed with qualitative research principles to evaluate parts that have been developed in daily practice [39]. Also some elements of participatory action research were used such as close collaboration or even between the researcher and participants. In this study, the main researcher also works as a COP oncology and also actively participated in the expert group and the discussion rounds. This ensures that our model does justice to practice. Second, our study is based on daily care practice and includes a heterogeneous group of patients, without strict exclusion criteria such as whether or not having a job, the nature, stage or phase of the disease. Finally, this study included patients with all kind of wishes in terms of work and income.

### Limitations

This study has a number of limitations. First, the care model was described based on the situation in one university hospital and based on experiences with a few patient groups. It is not known whether this care model could also be appropriate in a different hospital context, in particular because the work-oriented care was developed in a participatory manner at the request of healthcare professionals and patients. Although the model has been implemented only to a limited extent, at this moment initiatives have been taken from healthcare, government and politics to regulate the broad accessibility of work-oriented medical care for the near future. Based on plausibility, it can be assumed that the care model can also apply elsewhere. Follow-up research will have to show whether this model needs adjustments.

Second, the condition for our care model is that the healthcare professional questions his patient about quality of life and pays special attention to work. This presupposes a paradigm shift in the task conception of healthcare professionals. An aspect that needs attention is the time of referral to the COP. Patients seem to be ‘randomly’ referred, i.e. when the patient actively raises a question or problem about work or when the nurse/nursing specialist actively requests it. This could mean that patients problems and questions are missed. A third weakness is the limited participation of patients in the development of the care model. We chose to do this because we felt that we do not have sufficient insights into how the processes in the hospital are organised. In both the expert group as well as the discussion rounds, however, it has been expressly requested to better bring in the patients perspective.

## Implications for Practice and Research

The next step for securing integrated work-oriented care is implementation and evaluation of our developed care model into the care pathways for other chronic conditions [41, 42]. Healthcare professionals are learning when to refer, however when no MDT has been set up, they are more often referred (too) late. Perhaps including a structured work-oriented triage systems in the care pathways could help [28, 43]. Targeted training for nurses/nursing specialists and medical specialists may contribute to identification or recognition of work problems [27, 28]. This may induce adequate referral to the COP, the drawing up of an intervention plan for non-complex requests for help, and guidance and monitoring of patients in achieving their goals [28]. Future research needs to focus on which role and part of the work-oriented care model suits the nursing domain and how this can be best put into practice [28, 36].

It is also important to expand our model to transmural work-oriented health networks and to describe the entire care chain. This fits increasing interest and attention within the work and social domain, including occupational medicine, for the importance of personalised care [44]. Also, in insurance medicine, research is beginning to join the trend of better organising care around the patient [45]. All of this is to better meet the explicit desire of patients to be able to address the topic of work in the regular care processes [30, 46].

Patient Reported Outcome Measures (PROMs) are used to map health (problems). Measuring PROMS’s can make quality of care transparent, but at present PROMs lack societal participation as an outcome measure.

Patient Reported Experience Measures (PREMs) are questionnaires about patient experiences of the care process. Work and societal participation should be added in both PROMs and PREMs. New outcome measures on societal participation will have to be developed for setting up impact assessments and measuring the quality of care.

## Conclusions

We developed a WMCM for the intramural part of clinical care in which (1) biomedical and biopsychological models were combined together with (2) the goals and wishes of the patient, and in which (3) a WoIP becomes part of the treatment and care plan for the patient.

## Data Availability

No datasets were generated or analysed during the current study.
